# Optimal allocation of HIV prevention funds for state health departments

**DOI:** 10.1371/journal.pone.0197421

**Published:** 2018-05-16

**Authors:** Emine Yaylali, Paul G. Farnham, Stacy Cohen, David W. Purcell, Heather Hauck, Stephanie L. Sansom

**Affiliations:** 1 Division of HIV/AIDS Prevention, National Center for HIV, Viral Hepatitis, STD and TB Prevention (NCHHSTP), Centers for Disease Prevention and Control (CDC), Atlanta, GA, United States of America; 2 HIV/AIDS Bureau, Health Resources and Services Administration, Rockville, MD, United States of America; Emory University School of Public Health, UNITED STATES

## Abstract

**Objective:**

To estimate the optimal allocation of Centers for Disease Control and Prevention (CDC) HIV prevention funds for health departments in 52 jurisdictions, incorporating Health Resources and Services Administration (HRSA) Ryan White HIV/AIDS Program funds, to improve outcomes along the HIV care continuum and prevent infections.

**Methods:**

Using surveillance data from 2010 to 2012 and budgetary data from 2012, we divided the 52 health departments into 5 groups varying by number of persons living with diagnosed HIV (PLWDH), median annual CDC HIV prevention budget, and median annual HRSA expenditures supporting linkage to care, retention in care, and adherence to antiretroviral therapy. Using an optimization and a Bernoulli process model, we solved for the optimal CDC prevention budget allocation for each health department group. The optimal allocation distributed the funds across prevention interventions and populations at risk for HIV to prevent the greatest number of new HIV cases annually.

**Results:**

Both the HIV prevention interventions funded by the optimal allocation of CDC HIV prevention funds and the proportions of the budget allocated were similar across health department groups, particularly those representing the large majority of PLWDH. Consistently funded interventions included testing, partner services and linkage to care and interventions for men who have sex with men (MSM). Sensitivity analyses showed that the optimal allocation shifted when there were differences in transmission category proportions and progress along the HIV care continuum.

**Conclusion:**

The robustness of the results suggests that most health departments can use these analyses to guide the investment of CDC HIV prevention funds into strategies to prevent the most new cases of HIV.

## Introduction

An estimated 1.2 million people in the United States are living with HIV infection and about 40,000 infections are diagnosed each year. Although the number of new diagnoses decreased 19% from 2005 to 2014, much of the progress has been limited to heterosexuals (a 35% decrease in diagnoses) and persons who inject drugs (a 63% decrease) compared with gay, bisexual, and other men who have sex with men (MSM), who have experienced a 6% increase[1]. New diagnoses among MSM, particularly younger men and black and Hispanic men, increased over the decade[1].

State and local health departments receive public funds, primarily from the Centers for Disease Control and Prevention (CDC), to deliver comprehensive, high-impact HIV prevention programs. In 2014, CDC provided $560 million in HIV prevention funding to health departments [2]. In 2012, the Health Services and Resources Administration (HRSA) Ryan White HIV/AIDS Program Parts A and B spent approximately $366 million for services that link persons living with HIV (PLWH) to care, sustain them in care, and promote adherence to antiretroviral therapy (ART)[3]. Some health departments also received state and local HIV prevention funding for these activities[4].

Health departments have sought guidance in how to most effectively allocate their CDC prevention funds across populations and interventions. In previous work, we found that the optimal allocation of CDC prevention funds was similar for 4 state and local health departments that participated in a pilot program for an HIV resource allocation tool[5]. That study included an evaluation of health department experience using the tool. The pilot sites faced varying degrees of challenges using the model, including assembling the necessary site-specific data inputs. Given this experience, we realized we needed a model that incorporated important heterogeneities, but that was useful for health departments, particularly those with smaller budgets and staff dedicated to HIV prevention. We wanted to explore whether the results from a model that did not rely on potentially difficult-to-obtain local data would provide insights for most health departments.

In this study, we explore the extent to which the optimal allocation varies across state health departments in the United States (50 states, the District of Columbia, and Puerto Rico). Given that HIV prevalence is the primary factor that varies across the jurisdictions, we grouped the 52 US jurisdictions into 5 prevalence groups. In addition to CDC HIV prevention funds, we included, but did not optimize, HRSA Ryan White HIV/AIDS Program Part A and B funds that were spent for services that improve linkage to care, retention in care, and adherence to ART. We used sensitivity analysis to determine whether and how the optimal allocation of CDC funding might vary with changes in proportions of people living with diagnosed HIV by transmission category and progress along the HIV care continuum.

## Methods

### Health department stratification

We rank ordered the 52 health departments by the average number of persons living with diagnosed HIV according to 2010–2012 CDC surveillance data[6]. We then divided the health departments into 5 groups of 10 to 11 each according to the number of people living with diagnosed HIV in their jurisdiction: low-, low-to-moderate-, moderate-, moderate-to-high- and high-prevalence groups. For each group, we calculated the median number of people living with diagnosed HIV among all the health department jurisdictions in the group[6]. The moderate-, moderate-to-high- and high-prevalence groups combined included 95% of persons living with diagnosed HIV in the United States. The low-to-moderate prevalence group included 4% of all people living with diagnosed HIV, and the low-prevalence, 1% of all people living with diagnosed HIV. (Please see Table A in S1 Appendix for the list of health departments and their HIV prevalence group.)

We calculated the median annual CDC budget provided to each group based on the 2012 CDC state and local health department grants for HIV prevention[2] (Table 1). We also calculated the median Ryan White HIV/AIDS Program Parts A and B expenditures among state and local health departments for linkage to care, retention in care, and adherence to ART based on the 2012 Grantee Expenditure Reports of the Ryan White HIV/AIDS Program Parts A and B[3].

**Table 1:** Median number of persons living with diagnosed HIV, proportion of persons living with diagnosed HIV (PLWDH), median CDC HIV prevention budget and HRSA expenditures supporting linkage to care, retention in care, and adherence to ART by group.

Group	Median number of persons living with diagnosed HIV(6–8)	Proportion of PLWDH to total PLWDH (6–8)	Median CDC HIV prevention budget (2), $	Median HRSA expenditures supporting linkage to care (3), $	Median HRSA expenditures supporting retention in care (3), $	Median HRSA expenditures supporting adherence to ART(3), $	Median HRSA expenditures supporting HIV care continuum (3), $
**Low**	609	1%	1,196,820	54,623	54,623	50,794	160,040
**Low-to-moderate**	2,592	4%	1,815,535	197,188	213,315	206,182	616,685
**Moderate**	8,727	9%	2,915,741	1,276,569	1,130,701	851,150	3,258,420
**Moderate-to-high**	15,202	17%	6,068,568	2,247,912	2,064,523	1,665,485	5,977,920
**High**	36,878	69%	13,236,577	5,304,971	4,982,027	4,673,036	14,960,035

To estimate the average annual number of people living with diagnosed HIV by transmission group and gender in each health department group, we applied the national percentages of these variables to the median total number of people living with diagnosed HIV in each health department group (Table B in S1 Appendix)[6–8]. Similarly, we applied 2012 national percentages of the distribution of all persons living with HIV along the HIV care continuum to each health department group (Table C in S1 Appendix)[9, 10].

### Model overview

In addition to the 3 HIV care continuum-related interventions—linkage to care, retention in care, and adherence to ART—the model also included testing in clinical and non-clinical settings, partner services, and behavioral interventions for persons living with and at high risk of acquiring HIV. Partner services resulted in new diagnoses. Behavioral interventions included individual- and group-level approaches to reduce risky sexual behaviors. Some of these interventions were targeted to a particular transmission group—MSM, persons who inject drugs (PWID), and heterosexuals (HET)—resulting in 16 combinations of interventions and populations.

The model combined a Bernoulli process model with an optimization model. Details of the model’s formulation can be found in the S1 Appendix and previous publications[5, 11]. Specific inputs are reported in Table 2. We used the Bernoulli process model to calculate the difference in the annual HIV transmission or acquisition risk with and without each intervention. Based on that difference, and intervention costs, including expected ART treatment costs, we calculated the incremental cost per new infection prevented compared with the baseline/status quo. The optimization model allocated the median CDC HIV prevention budget for each health department group across all interventions, given each intervention’s cost-effectiveness. The model allocated funds from the most to the least cost-effective interventions until: (i) the budget was exhausted, or (ii) all interventions were funded to their maximum capacity. We defined maximum capacity as the proportion of the target population that was reachable by an intervention in a single year, considering program constraints and individuals’ willingness to participate. The annual proportions of eligible people who could be reached were assumed to be as follows: for testing in clinical and non-clinical settings, 10%; for partner services, 5%; for linkage, retention, and adherence programs, 20%; for behavioral interventions for persons living with HIV, 20%; and for behavioral interventions for those at risk of acquiring HIV, 10%. The proportions were based on an analysis of the City of Philadelphia’s CDC HIV prevention budget and the consensus of modelers and public health practitioners.

**Table 2:** Input parameters.

Parameter	Value	Source
Per-act HIV transmission probability, %		
Vaginal insertive	0.04	[12]
Vaginal receptive	0.08	[12]
Anal insertive	0.11	[13, 14]
Anal receptive	0.82	[15]
Needle-sharing injection drug use	0.30	[16]
Annual number of sex acts, all partners	68	[17, 18]
Annual number of injections, all partners	300	[19]
Annual number of partners		
Heterosexuals	1.1	[20]
PWID	2.5	[21]
MSM	3.68	[22]
Proportion with more than 1 concurrent positive partner, %		
Heterosexuals	5	[23]
PWID	19.35	[24]
MSM	19.35	[24]
Proportion of sex acts protected by condoms, %		
Heterosexuals	20.15	[25]
PWID	20.15	[25]
MSM	50	[26]
Proportion of injections in which needles are shared among users,%	5	[19]
Intervention efficacy, %		
Reduction in HIV transmission because of viral load suppression	96	[27]
Condom effectiveness in reducing HIV transmission	80	[28]
Reduction in needle sharing transmission because of viral load suppression	50	[29]
Reduction in condomless sex prevalence among positive aware persons because of testing	53	[30]
Reduction in needle sharing because of testing	26.5	Assumed to be half of the reduction in unprotected sexual intercourse
Reduction in condomless sex acts among HIV-positive persons because of behavioral interventions	27	[31–33]
Reduction in condomless sex acts among HIV-negative persons because of behavioral interventions	12	[34, 35]
Intervention Cost (2012$)		
Cost per test in clinical settings	31	[36–38]
Cost per test in nonclinical settings	138	[36, 37, 39]
Cost per partner tested and notified of a new positive diagnosis	7,737	[36, 40]
Cost per additional patient linked to care	4,825	[41]
Cost per additional client retained in care	1,900	[42, 43]
Cost per additional patient put on an intervention to improve the adherence to ART	3,551	[44–46]
Cost per client served in the behavioral intervention for positives	1,544	[36]
Cost per client served in the behavioral intervention for negatives	879	[36, 47]
Sero-prevalence, %		
Sero-prevalence of HIV: testing in clinical settings	0.60	[37, 48, 49]
Sero-prevalence of HIV: testing in non-clinical settings		
HET	0.51	[36]
PWID	1.50	[36]
MSM	3.00	[36]
Duration of intervention effect (years)		
HIV testing (clinical, non-clinical) and partner services	5	Assumption
Linkage to care, retention in care, and adherence to ART	2	Assumption
Behavioral interventions	1	Assumption
Maximum reach, %		
HIV testing (clinical and non-clinical)	10	Assumption
Partner services	5	Assumption
Linkage to care, retention in care, and adherence to ART	20	Assumption
Behavioral interventions for HIV-infected persons	20	Assumption
Behavioral interventions for HIV-uninfected persons	10	Assumption
Treatment Cost (2012$)		
Annual ART cost	16,005	[50]

The model also incorporated HRSA Ryan White HIV/AIDS Program Parts A and B funding expended by each health department group for services related to linkage to care, retention in care, and adherence to ART. We held HRSA Ryan White HIV/AIDS Program Parts A and B expenditure amounts constant and optimized only CDC HIV prevention funds.

### Outcomes

We first calculated the incremental cost per case of HIV prevented compared with the baseline/status quo for all interventions. Based on those cost-effectiveness estimates, we reported the optimal allocation of the CDC prevention budget among interventions and persons living with or at risk for HIV for the 5 health department groups, holding the HRSA Ryan White HIV/AIDS Program Parts A and B expenditures constant. Under the optimal CDC budget allocation for each health department group, we calculated the expected number of HIV infections prevented compared with the baseline/status quo, and the average budget per case prevented. We also reported the annual budget per person living with diagnosed HIV.

### Scenario analyses

We analyzed how changes in a state-specific HIV transmission-group profile might affect the optimal allocation by identifying 3 states where the proportion of persons living with HIV by transmission group varied most substantially from the national average. Those states were California (higher-than-average representation of MSM among people living with HIV), Florida (higher-than-average representation of heterosexuals among people living with HIV), and New Jersey (higher-than-average representation of PWID among people living with HIV) (Table D in S1 Appendix). All 3 states were from the high-prevalence health department group, which allowed us to compare the 3 state-specific allocations with the allocation for the entire group. In scenario analysis, we calculated the optimal allocation of CDC funds separately for each of the 3 states using state-specific CDC budgets and HRSA expenditures, as well as state-specific data for the number of people living with diagnosed HIV and their stratification by transmission group and gender[2, 3, 6–8]. We compared this optimal allocation and corresponding outcomes to those under the optimal allocation for the high-prevalence group applied to state-specific characteristics including state-specific CDC budgets and HRSA expenditures, transmission-group profile, and number of people living with diagnosed HIV.

We also determined the effect on the optimal allocation of state-specific differences in the proportions of persons living with HIV who had achieved various stages along the HIV care continuum. For this analysis, we used the high-prevalence health department group as our base case for brevity and because the majority of people living with diagnosed HIV belong to this group. We conducted a one-way sensitivity analysis on 2 HIV care continuum parameters: the proportion of undiagnosed HIV-infected persons among all PLWH and the proportion of diagnosed HIV-infected persons who achieved viral load suppression (VLS) (Table E in S1 Appendix). We focused on these two parameters because of their large association with HIV transmission. We varied these parameters +/-50% or as their bounds dictated by the interdependence of the continuum-of-care parameters. For example, in the model, the proportion of diagnosed HIV-infected persons who achieve VLS could not be greater than the proportion of diagnosed HIV-infected persons who are prescribed ART.

## Results

The incremental cost per case of HIV prevented compared with the baseline/status quo varied substantially by intervention and population (Table 3). Testing in non-clinical settings for MSM ($130,509) and partner services for MSM ($130,639) were the most cost-effective interventions and behavioral interventions for HIV-negative PWID ($958,355) and heterosexuals ($115,592,270), the least.

**Table 3:** Incremental cost per case of HIV prevented compared with the baseline/status quo by intervention.

Interventions	Incremental cost per new case prevented, $
**Testing in nonclinical settings: MSM**	130,509
**Partner services: MSM**	130,696
**Behavioral intervention for HIV+: MSM**	151,413
**Testing in clinical settings**	185,251
**Linkage to care**	246,411
**Adherence to ART**	246,454
**Partner services: PWID**	299,805
**Behavioral intervention for HIV-: MSM**	327,128
**Testing in nonclinical settings: PWID**	350,097
**Partner services: HET**	405,358
**Behavioral intervention for HIV+: HET**	430,261
**Retention in care**	433,150
**Behavioral intervention for HIV+: PWID**	489,909
**Testing in nonclinical settings: HET**	738,452
**Behavioral intervention for HIV-: PWID**	958,355
**Behavioral intervention for HIV-: HET**	115,592,270

Once the HRSA Ryan White HIV/AIDS Program Parts A and B expenditures were taken into account for services related to linkage to care, retention in care, and adherence to ART, the model optimally allocated CDC funds to the same 7 most cost-effective interventions in all 5 health department groups (Table 4). Those interventions included: testing in non-clinical settings for MSM, partner services for MSM, behavioral interventions for MSM living with HIV, testing in clinical settings, partner services for PWID, and behavioral interventions for MSM at risk for acquiring HIV. The model also allocated a modest amount (3% to 12% of the budget) for linkage-to-care interventions, in addition to the HRSA funding. However, given the HRSA expenditures on services related to retention in care and adherence to ART, no CDC funding was allocated for these interventions, reflecting that CDC funds might be more cost-effectively spent on other interventions.

**Table 4:** Optimal allocation of CDC budget, allocation of HRSA Ryan White HIV/AIDS Program Parts A and B expenditures, and other model results by health department group.

	Optimal allocation of CDC budget, $ HRSA Ryan White HIV/AIDS Program Parts A and B expenditures supporting continuum of care, $									
Intervention	Low		Low-to-moderate		Moderate		Moderate-to-High		High	
**Testing in nonclinical settings: MSM**	33,173	3%*	141,131	8%	475,086	16%	827,597	14%	2,007,686	15%
**Partner services: MSM**	16,728	1%	71,168	4%	239,569	8%	417,328	7%	1,012,405	8%
**Behavioral intervention for HIV+: MSM**	45,515	4%	193,637	11%	651,835	22%	1,135,492	19%	2,754,616	21%
**Testing in clinical settings**	66,144	6%	281,401	15%	947,271	32%	1,650,140	27%	4,003,112	30%
**Linkage to care**	43,829 54,623	4%	221, 664 197,188	12%	133,401 1,276,569	5%	208,247 2,247,912	3%	653,481 5,304,971	5%
**Adherence to ART**	0 31,317		0 133,236		0 448,510		0 781,301		0 1,895,377	
**Partner services: PWID**	5,008	0.4%	21,307	1%	71,725	2%	124,944	2%	303,106	2%
**Behavioral intervention for HIV-: MSM**	253,266	21%	885,227	49%	396,852	14%	1,704,820	28%	2,502,171	19%
**Testing in nonclinical settings: PWID**	19,864	2%								
**Partner services: HET**	8,423	1%								
**Behavioral intervention for HIV+: HET**	22,918	2%								
**Retention in care**	0 54,507		0 213,315		0 780,620		0 1,359,835		0 3,298,855	
**Behavioral intervention for HIV+: PWID**	13,627	1%								
**Testing in nonclinical settings: HET**	98,258	8%								
**Behavioral intervention for HIV-: PWID**	56,323	5%								
**Behavioral intervention for HIV-: HET**	513,743	43%								
**CDC budget allocated, $**	**1,196,820**		**1,815,535**		**2,915,741**		**6,068,568**		**13,236,577**	
**HRSA Ryan White HIV/AIDS Program Parts A and B expenditures, $**	**140,448**		**543,739**		**2,505,700**		**4,389,048**		**10,499,203**	
**CDC budget + HRSA Ryan White HIV/AIDS Program Parts A and B expenditures allocated, $**	**1,337,268**		**2,359,274**		**5,421,441**		**10,457,616**		**23,735,780**	
**Infections prevented**	**3**		**10**		**26**		**48**		**111**	
**Budget per HIV-infected person, $**	**2,195**		**910**		**621**		**688**		**644**	
**Average budget per case of HIV prevented, $**	**471,189**		**237,499**		**211,048**		**218,564**		**213,684**	

* Percent of CDC budget allocated to each intervention

The proportion of the CDC budget allocated across those 7 interventions in the 3 higher groups (moderate, moderate-to-high, and high) was similar. In the low-to-moderate group, the allocation also went toward the same 7 interventions, but nearly 50% of the allocation went to behavioral interventions for MSM at risk for HIV compared to 14–28% in the higher prevalence groups. In the low-prevalence group, the allocation was spread across all 16 interventions, with the highest level of funding for behavior intervention for heterosexuals at risk for HIV, the least cost-effective intervention.

Under the optimal allocation, the average annual number of infections prevented varied from 3 to 111 infections (Table 4). The average budget (including HRSA expenditures) per case of HIV prevented was similar for the 4 health department groups with the highest HIV prevalence ($211,000 to $237,000), but was more than double for the group with the lowest HIV prevalence ($471,000). As a comparator, the 2012 estimated lifetime treatment cost of HIV is $416,977[51].

The optimal CDC allocation for health departments with substantially different transmission-group profiles from the national average resulted in larger allocations to some programs for those groups, with corresponding reductions to other interventions (Table 5). In California, a state with a larger proportion of MSM than average, a larger proportion of funds was allocated to testing MSM and behavioral programs for MSM living with HIV, while a smaller proportion of funds went to testing all people in clinical settings and behavioral programs for MSM at risk for HIV. This shift resulted in a 3% increase in cases prevented (407) than from application of the overall allocation for the high-prevalence group of states (397). In Florida, a state with a larger proportion of heterosexuals, a larger proportion of funds was allocated toward testing in clinical settings, a more cost-effective approach for diagnosing HIV among heterosexuals than testing heterosexuals in non-clinical settings. A larger proportion was also allocated to linkage-to-care interventions. A smaller proportion of CDC funds went to behavioral programs for MSM at risk for HIV. This shift resulted in a 2% increase in cases prevented. In New Jersey, a state with a larger proportion of PWID than average, funding for the testing of MSM in non-clinical settings and of all persons in clinical settings remained constant, while funding for partner services among PWID slightly increased. Funding for behavioral interventions for MSM at risk for HIV exceeded 50% in both scenarios, either because the maximum number of people who inject drugs had been reached by other interventions, or other interventions that target PWIDs were less cost-effective than behavioral programs for MSM at risk for HIV. Because of those reasons, there was little difference (<1%) in New Jersey in the number of HIV cases prevented using the allocation for all high-prevalence health departments versus an allocation specific to New Jersey’s transmission-group profile.

**Table 5:** Optimal CDC HIV prevention funding allocation for California, Florida and New Jersey: Allocation for high prevalence group versus state-specific characteristics.

Intervention	California (1)	California (2)	Florida (1)	Florida (2)	New Jersey (1)**	New Jersey (2)
**Testing in nonclinical settings: MSM**	15%	19%	15%	16%	8%	8%
**Partner services: MSM**	8%	10%	8%	8%	4%	4%
**Behavioral intervention for HIV+: MSM**	21%	26%	21%	21%	10%	10%
**Testing in clinical settings**	30%	27%	30%	35%	23%	23%
**Linkage to care**	5% + H*	H	5% + H	7% + H	H	H
**Adherence to ART**	H	H	H	H	H	H
**Partner services: PWID**	2%	1%	2%	2%	2%	3%
**Behavioral intervention for HIV-: MSM**	19%	17%	19%	11%	53%	52%
**Testing in nonclinical settings: PWID**	-	-	-	-	-	-
**Partner services: HET**	-	-	-	-	-	-
**Behavioral intervention for HIV+: HET**	-	-	-	-	-	-
**Retention in care**	H	H	H	H	H	H
**Behavioral intervention for HIV+: PWID**	-	-	-	-	-	-
**Testing in nonclinical settings: HET**	-	-	-	-	-	-
**Behavioral intervention for HIV-: PWID**	-	-	-	-	-	-
**Behavioral intervention for HIV-: HET**	-	-	-	-	-	-
**CDC budget allocated, $**	**42,959,267**	**42,959,267**	**28,707,706**	**28,707,706**	**16,903,650**	**16,903,650**
**HRSA Ryan White HIV/AIDS Program Parts A and B expenditures, $**	**32,276,786**	**32,276,786**	**26,205,345**	**26,205,345**	**10,866,390**	**10,866,390**
**Infections prevented**	**397**	**407**	**243**	**247**	**105**	**105**
**Average budget per case of HIV prevented, $**	**189,668**	**184,676**	**225,973**	**221,880**	**265,021**	**264,912**

(1) Indicates the optimal CDC prevention fund allocation for the high prevalence group applied to state-specific characteristics

(2) Indicates the optimal CDC prevention fund allocation for state-specific characteristics, including budget, number of people living with diagnosed HIV, and transmission-group profile.

*: H indicates HRSA Ryan White HIV/AIDS Program Parts A and B Expenditures

**: The optimal allocation for New Jersey differed from the high prevalence allocation applied to California and Florida. In New Jersey, the optimal allocation for the high prevalence group allocated more funding for some categories of interventions than there were people to serve. For example, the optimal high prevalence allocation spent 15% of the budget to testing MSM in non-clinical settings, but given the number of MSM available for testing in New Jersey, only 8% of that state’s CDC prevention budget was needed to provide the testing.

The optimal CDC allocation also changed when the proportion of persons diagnosed with HIV or with viral load suppression changed substantially from the base case (Table 6). When the proportion of undiagnosed HIV-infected persons was decreased to its lower bound (6.4%, compared to a base case of 12.8%), allocations to testing and partner services interventions decreased and the remaining CDC HIV prevention funding was distributed to behavioral interventions for high-risk MSM. When the proportion of undiagnosed HIV-infected persons was increased to its upper bound of 19.2%, funding increased for the most cost-effective testing strategies. Similarly, when the proportion of persons diagnosed with HIV who are virally suppressed was decreased to 20.9% from the base case of 41.7%, the model allocated CDC funding for ART adherence interventions. When the proportion virally suppressed was increased to 50%, CDC funding shifted away from adherence interventions to the previously unfunded testing in non-clinical settings of PWID.

**Table 6:** Optimal CDC HIV prevention funding allocation: Varying the proportion of PLWH who are diagnosed with HIV and who have achieved viral load suppression (VLS) in the high health department group.

Intervention	High	% Undiagnosed LB (6.4%)	% Undiagnosed UB (19.2%)	% VLS LB (20.9%)	% VLS UB (50.0%)
**Testing in nonclinical settings: MSM**	15%	8%	23%	15%	15%
**Partner services: MSM**	8%	4%	11%	8%	8%
**Behavioral intervention for HIV+: MSM**	21%	22%	19%	21%	21%
**Testing in clinical settings**	30%	15%	45%	30%	30%
**Linkage to care**	5%+H*	8% + H	1% + H	H	5%+H
**Adherence to ART**	H	H	H	15% +H	-
**Partner services: PWID**	2%	1%	-	-	2%
**Behavioral intervention for HIV-: MSM**	19%	42%	-	11%	10%
**Testing in nonclinical settings: PWID**	-	-	-	-	9%
**Partner services: HET**	-	-	-	-	-
**Behavioral intervention for HIV+: HET**	-	-	-	-	-
**Retention in care**	H	H	H	H	H
**Behavioral intervention for HIV+: PWID**	-	-	-	-	-
**Testing in nonclinical settings: HET**	-	-	-	-	-
**Behavioral intervention for HIV-: PWID**	-	-	-	-	-
**Behavioral intervention for HIV-: HET**	-	-	-	-	-
**CDC budget allocated, $**	**13,236,577**	**13,236,577**	**13,236,577**	**13,236,577**	**13,236,577**
**HRSA Ryan White HIV/AIDS Program Parts A and B expenditures, $**	**10,499,203**	**10,880,431**	**10,117,975**	**13,276,863**	**8,603,827**
**Infections prevented**	**111**	**102**	**120**	**146**	**114**
**Average budget per case of HIV prevented, $**	**213,684**	**237,184**	**194,162**	**181,478**	**192,298**

H* indicates HRSA Ryan White HIV/AIDS Program Parts A and B Expenditures; LB = lower bound; UB = upper bound; VLS = viral load suppression

## Discussion

Our results show that the optimal allocation of CDC HIV prevention funds—both in terms of the interventions funded and the proportion of the CDC budget directed toward those interventions—is similar for the health departments serving the great majority of persons living with diagnosed HIV. Given HRSA expenditures on services for linkage to and retention in care, and adherence to ART, the optimal results support investments in testing in non-clinical settings for MSM, partner services for MSM, behavioral interventions for MSM living with HIV, testing across populations in clinical settings, partner services for PWID, and behavioral interventions for MSM at risk for acquiring HIV. In the absence of a tool that could be used with local parameters, we believe these results are a good starting point to guide resource allocation decisions. Robustness of the results shows that there is a common set of policies, such as the importance of testing and care continuum interventions and targeting high-risk populations such as MSM, which can be applied in any HIV prevalence level.

Based on CDC surveillance reports[6–8] and the NCHHSTP Atlas[52], we estimated that the transmission category of persons living with diagnosed HIV at the state level usually approximates the profile of transmission categories nationally. However, health departments serving communities where the transmission category profile varies substantially from the national average might adjust their CDC budget allocation accordingly to provide more funding to disproportionately represented transmission groups, which could yield better health outcomes. In California, with its larger population of MSM, for example, our scenario analysis shifted more CDC funding to testing of MSM and behavioral interventions for MSM diagnosed with HIV, and this strategy led to more cases prevented. We note that there are 2 other strategies health departments might consider to shift the optimal allocation of funds. First, they might develop more cost-effective approaches to deliver a specific intervention, compared with the cost-effectiveness estimates we used in this analysis. For instance, in New Jersey, with its higher-than-average representation of persons who inject drugs among all persons diagnosed with HIV, the ranking of the cost-effectiveness of testing in non-clinical settings persons who inject drugs falls too low in our analysis to receive funding. However, if program managers who implement testing in non-clinical settings found ways to deliver testing more efficiently, this intervention would become a more viable option. Alternatively, program directors might seek to efficiently expand programs, such as partner services for persons who inject drugs. In our analysis, and based on historical data, only 5% of persons who inject drugs could be reached by partner services [5]. It may be possible to expand these services in New Jersey without incurring large additional costs.

Similarly, state-level variations in the proportions of persons living with HIV at different points along the HIV care continuum may warrant adjustments in the optimal CDC funding allocation to address those variations. In particular, health departments may want to consider adjusting their allocations to testing for HIV depending on the proportion of PLWH estimated to be undiagnosed. And they may want to adjust their allocations to ART adherence programs based on levels of viral load suppression observed among those diagnosed.

In our previous work examining the optimal allocation of CDC HIV prevention funds for 4 state and local health departments, the model emphasized testing the general population in clinical settings, testing MSM and PWID in non-clinical settings, and, usually, interventions associated with linkage to care, retention in care, and adherence to ART. The optimal allocations included no funding for behavioral interventions. In this analysis, when we looked across the 5 broad groups of health departments and included the HRSA Ryan White HIV/AIDS Program Parts A and B expenditures that support the 3 continuum-related interventions, emphasis remained on CDC funding of testing the general population in clinical settings and MSM in non-clinical settings. However, consideration of the HRSA Ryan White HIV/AIDS Program Parts A and B expenditures led to reductions in CDC funding for linkage to care interventions and the elimination of CDC funding for retention in care and adherence to ART interventions. Instead, CDC funds were directed toward behavioral interventions, usually for MSM. Our findings suggest that close collaboration within health departments to allocate funds from different sources might lead to more efficiencies. In fact, both HRSA and CDC have supported collaborations between or integration of local CDC and HRSA planning bodies as prevention and treatment efforts have increasingly overlapped.

Among the 5 groups of health departments, the optimal CDC allocation differed most markedly among the low-prevalence and low-to-moderate prevalence groups. This difference can largely be explained by the limited size of the populations eligible for HIV prevention interventions (i.e., 5% of persons living with diagnosed HIV are represented by these 2 groups), and our assumptions about the maximum proportion of the population that can be reached by an intervention. For example, in the low-to-moderate prevalence group, the optimal allocation funded the 6 most cost-effective interventions with 1% to 15% of the budget each, and then allocated 49% of the budget into the 7^th^ most cost-effective intervention, behavioral programs for MSM at risk of acquiring HIV. This result occurred because the preceding interventions reached the maximum number of people possible, but the size of the at-risk MSM group was sufficient to absorb the remainder of the funding. Similarly, due to the very low numbers of people to serve in the low-prevalence group, the optimal allocation provided funding to every intervention, and the least cost-effective intervention, behavioral programs for heterosexuals at risk for acquiring HIV, absorbed 43% of the budget. This explains why the combined CDC HIV prevention funding and HRSA expenditures per case prevented increased from about $211,000 to $237,000 among the 4 higher-prevalence groups and then jumped to $471,000 in the low-prevalence group. Health departments whose optimal allocations suggested funding the least cost-effective interventions may choose to see if they can find efficient ways to expand the maximum capacity of the most cost-effective interventions.

Our study has several limitations. We assumed the cost, efficacy, and maximum reach of interventions were the same across health department groups. However, these might vary depending on jurisdictional implementation practices, participant characteristics, and potential economies of scale. The intervention costs typically did not include program setup and administrative costs, which could result in fewer persons served by each intervention. We also excluded funding sources other than CDC and HRSA, such as local, state, and other federal sources; including these sources could result in the expansion of programs.

We did not include a number of recognized prevention strategies in the model. CDC funding for Syringe Services Programs (SSPs) is limited to activities and equipment other than the syringes and needles themselves, and all funding requires prior approval for each health department. Not all health departments are approved. It is still unclear how federal expenditures will affect injection-related transmissions. Because we could not associate funding for this intervention to specific reductions in shared syringe use or HIV incidence, we excluded it from this version of the model.

With respect to pre-exposure prophylaxis (PrEP), CDC funding is limited to screening for PrEP eligibility, linkage to PrEP services, support for PrEP adherence, and increasing consumer and provider knowledge of PrEP. It is as yet unclear how effectively these funds serve to get persons at high risk of acquiring HIV on PrEP and to maintain high adherence thereafter. However, ongoing federally-funded PrEP demonstration projects eventually could establish the association between federal funding for PrEP and reduced incidence.

The model also considers HIV care continuum interventions as discrete rather than combined interventions due to limited data on the effectiveness of combined interventions. Combined interventions have the potential to be less expensive and more effective than those delivered separately; future research to quantify their costs and effects would be useful. In addition, we lacked sufficient cost and efficacy data on interventional surveillance and on investigations of and responses to molecular transmission clusters, even though these are required under recent CDC funding instructions to health departments. In addition, the existing literature on the cost-effectiveness of partner services is limited to effectiveness in detecting previously undiagnosed infections among partners. We lack data to determine the effect of partner services on linking or relinking to care persons previously diagnosed with HIV. This is a potentially important benefit that warrants precise measurement.

Recommended prevention interventions change over time, along with best strategies for implementing them. Often, research lags on the costs and efficacy of those interventions and their implementation strategies. We generally take a conservative approach by excluding those interventions, rather than risk suggesting that scarce public funds be allocated to interventions and strategies with questionable efficacy. However, the process is dynamic, and to the extent robust scientific methods are used to evaluate the strength of evidence underscoring these new interventions and implementation approaches, we will incorporate them into future models. For now, we suggest the scientific community continue to evaluate these new approaches. CDC, for example, has funded a data-to-care trial where surveillance data are used for programmatic purposes as well as a demonstration project for health department use of molecular surveillance.

We have presented results from a resource allocation model for CDC’s HIV prevention funds that applies to health departments across the United States and that includes the HRSA Ryan White HIV/AIDS Program Parts A and B expenditures for services that support the HIV continuum of care. Our results showed that the optimal CDC allocation—in terms of interventions funded and the proportion of the CDC budget allocated—varied little among the majority of health departments and highlighted the importance of implementing and scaling up these seven key interventions. Our results also identified how adjustments to the funding allocation might be made to better match characteristics of local populations and local progress in engaging persons living with diagnosed HIV in care and treatment. These results have practical implications because most health departments should find them useful to help guide decisions about the optimal allocation of their CDC HIV prevention funds.

## Supporting information

S1 Appendix (DOCX)
