# Supplementary material for: Optimal allocation of HIV prevention funds for state health departments
Source: PLoS One. 2018 May 16;13(5):e0197421. doi: 10.1371/journal.pone.0197421 (PMC5955542; doi:10.1371/journal.pone.0197421)
Supplement: S1 Appendix — (DOCX) [file pone.0197421.s001.docx]

**APPENDIX**

In this appendix, we present additional data on model inputs: the classification of the states by 5 health department categories; data used to derive the number of persons in the transmission categories and along the continuum of care; inputs for the Bernoulli model and optimization model; and sensitivity analysis ranges. We also present the optimization model formulation and formulas for the model outcomes.

Health department category specific model inputs:

Table A: Classification of 50 states, District of Columbia, and Puerto Rico by prevalence category (1-3)

| Low  HIV prevalence areas (n=11) | Low-to-moderate HIV prevalence areas (n=10) | Moderate  HIV prevalence areas (n=10) | Moderate-to-high HIV prevalence areas (n=10) | High  HIV prevalence areas (n=11) |
| --- | --- | --- | --- | --- |
| Alaska  Idaho  Maine  Montana  Nebraska  New Hampshire  North Dakota  South Dakota  Vermont  West Virginia  Wyoming | Arkansas  Delaware  Hawaii  Iowa  Kansas  New Mexico  Oklahoma  Oregon  Rhode Island  Utah | Colorado  Connecticut  Indiana  Kentucky  Minnesota  Mississippi  Missouri  Nevada  Washington  Wisconsin | Alabama  Arizona  District of Columbia  Louisiana  Massachusetts  Michigan  Ohio  Puerto Rico  South Carolina  Tennessee | California  Florida  Georgia  Illinois  Maryland  New Jersey  New York  North Carolina  Pennsylvania  Texas  Virginia |

Table B: Percent of persons living with diagnosed HIV by transmission group and gender, United States, 2010-2013

| Transmission Group/Gender | Persons living with diagnosed HIV(1-3)* |
| --- | --- |
| HET Female | 19% |
| HET Male | 9% |
| HET Total | 28% |
| PWID Female | 6% |
| PWID Male | 10% |
| PWID Total | 16% |
| MSM | 55% |

*: These percentages are calculated as the average annual number of PLWDH (2010-2013) in each category divided by total PLWDH

Table C: Percent distribution of persons living with HIV along the continuum-of-care, 2012

|  | Value, % | Source |
| --- | --- | --- |
| Percent undiagnosed HIV-infected persons among all PLWH | 12.8 | (4) |
| Percent diagnosed HIV-infected persons who are linked to care | 80.8 | (5) |
| Percent diagnosed HIV-infected persons who are retained in care | 53.8 | (4) |
| Percent diagnosed HIV-infected persons who are prescribed ART | 50.0 | (4) |
| Percent diagnosed HIV-infected persons who achieve viral load suppression | 41.7 | (4) |

Sensitivity Analysis:

Table D: Persons living with diagnosed HIV by transmission group and gender: California, Florida, New Jersey, and national average

|  | Persons living with diagnosed HIV(1) | | | |  |
| --- | --- | --- | --- | --- | --- |
|  | **National Avg.** | **California** | **Florida** | **New Jersey** | |
| HET F | 19% | 9% | 26% | 23% | |
| HET M | 9% | 4% | 15% | 11% | |
| HET Total | 28% | 13% | 41% | 33% | |
| PWID F | 6% | 3% | 4% | 11% | |
| PWID M | 10% | 6% | 6% | 19% | |
| PWID Total | 16% | 9% | 10% | 30% | |
| MSM | 55% | 78% | 49% | 37% | |

Table E: Sensitivity analysis: variation in the proportion of PLWH who are diagnosed with HIV and who have achieved viral load suppression compared with national averages

|  | **National Avg., %** | **Lower Bound,%** | **Upper Bound,%** |
| --- | --- | --- | --- |
| Percent undiagnosed HIV-infected persons among all PLWH | 12.8 | 6.4 | 19.2 |
| Percent diagnosed HIV-infected persons who achieve viral load suppression | 41.7 | 20.9 | 50.0* |

*: This parameter at maximum cannot exceed the proportion of diagnosed HIV-infected persons who are prescribed ART. See Table A.3.

| Table F. Sensitivity analysis of maximum reach assumptions | | | | |
| --- | --- | --- | --- | --- |
| **`** | **Optimal allocation of CDC budget, $**  **HRSA Ryan White HIV/AIDS Program Parts A and B expenditures supporting continuum of care, $** | | | |
| **Intervention (base case maximum reach)** | **High** | | **Maximum reach: 20% for all interventions** | |
| **Testing in nonclinical settings: MSM (10%)** | 2,007,686 | 15% | 4,015,372 | 30% |
| **Partner services: MSM (5%)** | 1,012,405 | 8% | 4,049,621 | 31% |
| **Behavioral intervention for HIV+: MSM (20%)** | 2,754,616 | 21% | 2,754,616 | 21% |
| **Testing in clinical settings (10%)** | 4,003,112 | 30% | 2,416,968 | 18% |
| **Linkage to care (20%)** | 653,481 5,304,971 | 5% | 0 5,304,971 |  |
| **Adherence to ART (20%)** | 0 1,895,377 |  | 0 1,895,377 |  |
| **Partner services: PWID (5%)** | 303,106 | 2% |  |  |
| **Behavioral intervention for HIV-: MSM (10%)** | 2,502,171 | 19% |  |  |
| **Testing in nonclinical settings: PWID (10%)** |  |  |  |  |
| **Partner services: HET (5%)** |  |  |  |  |
| **Behavioral intervention for HIV+: HET (20%)** |  |  |  |  |
| **Retention in care (20%)** | 0 3,298,855 |  | 0 3,298,855 |  |
| **Behavioral intervention for HIV+: PWID (20%)** |  |  |  |  |
| **Testing in nonclinical settings: HET (10%)** |  |  |  |  |
| **Behavioral intervention for HIV-: PWID (10%)** |  |  |  |  |
| **Behavioral intervention for HIV-: HET (10%)** |  |  |  |  |
| **CDC budget allocated, $** | **13,236,577** | | **13,236,577** | |
| **HRSA Ryan White HIV/AIDS Program Parts A and B expenditures, $** | **10,499,203** | | **10,499,203** | |
| **CDC budget + HRSA Ryan White HIV/AIDS Program Parts A and B expenditures allocated, $** | **23,735,780** | | **23,735,780** | |
| **Infections prevented** | **111** | | **130** | |
| **Budget per HIV-infected person, $** | **644** | | **644** | |
| **Average budget per case of HIV prevented, $** | **213,684** | | **182,583** | |

We replaced our base case assumptions about the maximum proportion of persons reachable each year by each intervention with an assumption that 20% could be reached annually, regardless of the type of intervention. The model increased allocations to the most cost-effective interventions and did not allocate to some of the interventions previously funded. Thus, the list of CDC-funded interventions shrank from 7 to 4 of which 3 were specifically for MSM living with HIV. Because more funds were going to more cost-effective interventions, the annual number of infections prevented increased 17% from 111 to 130, and the budget per case of HIV prevented also declined 15%, from $213,684 to $182,583.

Optimization Model:

The model formulation is summarized below:

$$\max\sum_{i} \sum_{j} \left( \frac{x_{ij}+ m_{ij}}{c_{ij}} \right) e_{ij}d_{i}$$

Subject to

$$\frac{{(x}_{ij}+m_{ij})}{c_{ij}}\leq f_{ij}N_{ij}$$

$\sum_{i} \sum_{j} x_{ij}\leq B$

$x_{ij}\geq0 \forall i, j$

where

Indices

$i$ HIV interventions where $i=1 to k$

$j$ Transmission group where $j=1 to h$

Decision variable

$x_{ij}$ Funding to allocate intervention $i$targeted at population $j$ where $i=1 to k$ and $j=1 to h$

Parameters

$B$ Amount of available funding to be allocated

$c_{ij}$ Average cost per effective outcome to provide an intervention $i$ in population $j$ to one client

$m_{ij}$ Funds restricted (“earmarked”) for intervention $i$ for population $j$ (exclusive of$B$)

$N_{ij}$ Number of individuals in population $j$ that are eligible for intervention $i$

$e_{ij}$ Expected annual number of potential infections averted per person served by intervention $i$ in population $j$

$d_{i}$ Duration of intervention $i$ effect

$f_{ij}$ Maximum percent of population reachable by intervention $i$ in population $j$

The objective of the model is to maximize the expected number of infections prevented over the duration of the intervention. If funding is earmarked for certain intervention(s), its effect is included in the objective function. The first constraint of the above model limits the amount of the budget allocated to an intervention to be less than or equal to the amount that is needed to serve the maximum percent of the population reachable by that intervention. We assumed that there would be a part of the target population that could never be reached. There is also a harder-to-reach population due to the limited budget, feasibility, and scalability of the intervention. Thus, we included the parameter f_ij_, the maximum percent of the population reachable by an intervention i in population j, in our model. Due to data unavailability and lack of relevant literature, we estimated these parameters from expert opinion, and we set them consistently among populations but varied them by intervention. Based on the previous analysis,(6, 7) we set the maximum reach at 20% for continuum-of-care interventions and behavioral interventions for HIV-infected persons, 10% for testing and behavioral interventions for HIV-uninfected persons, and 5% for partner services. The second constraint of the model ensures that the allocated funding does not exceed the available budget.

Model Outcomes:

1. Incremental cost per case of HIV prevented compared with the baseline/status quo:

The inverses of the coefficients in the objective function are equivalent to the incremental cost per case of HIV prevented compared with the baseline/status quo by intervention. We applied a 3% discount rate if the duration of intervention effect is greater than 1 year. This is formulated as follows:

$\frac{c_{\mathrm{ij}}}{e_{ij}d_{i}}$for intervention i for transmission group j

1. Average budget per case of HIV prevented

We estimated the average budget per case of HIV prevented by dividing the total budget allocated (CDC and HRSA) by the expected number of HIV infections prevented, which is a model outcome for each category. This is formulated as follows:

$\frac{{Total CDC budget allocated}_{k}+{Total HRSA budget allocated}_{k}}{{Total HIV infections prevented}_{k}}$ for category *k*

1. Budget per HIV-infected person

We measured the budget per HIV-infected person by dividing the total available CDC and HRSA budget for each category by the median number of persons living with diagnosed HIV. This is formulated as follows:

$\frac{{Total CDC budget available}_{k}+{Total HRSA budget available}_{k}}{{Median number of persons living with diagnosed HIV}_{k}}$ for category *k*

If the maximum reach constraint is binding and interventions are funded with less than the available budget, then the allocated budget (ii above) would be less than the available budget. Otherwise, both are the same.

1. Centers for Disease Control and Prevention. HIV Surveillance Report, 2013. February 2015.

2. Centers for Disease Control and Prevention. HIV Surveillance Report, 2012. November 2014.

3. Centers for Disease Control and Prevention. HIV Surveillance Report, 2011. February 2013.

4. Centers for Disease Control and Prevention. Monitoring selected national HIV prevention and care objectives by using HIV surveillance data—United States and 6 U.S. dependent areas—2013 July 2015. Contract No.: 2.

5. Centers for Disease Control and Prevention. Monitoring selected national HIV prevention and care objectives by using HIV surveillance data—United States and 6 U.S. dependent areas—2012 November 2014. Contract No.: 3.

6. Lin F, Farnham PG, Shrestha RK, Mermin J, Sansom SL. Cost Effectiveness of HIV Prevention Interventions in the U.S. American Journal of Preventive Medicine. 2016; 50: 699-708.

7. Yaylali E, Farnham PG, Schneider KL, Landers SJ, Kouzouian O, Lasry A, et al. From Theory to Practice: Implementation of a Resource Allocation Model in Health Departments. Journal of Public Health Management and Practice. 2016; 22: 567-575.
